# The role of firearm and alcohol availability in firearm suicide: A population-based weighted case-control study

**DOI:** 10.1016/j.healthplace.2023.102969

**Published:** 2023-01-19

**Authors:** Veronica A. Pear, Garen J. Wintemute, Nicholas P. Jewell, Jennifer Ahern

**Affiliations:** a Violence Prevention Research Program, Department of Emergency Medicine, University of California, Davis School of Medicine, USA; b Division of Epidemiology, University of California, Berkeley School of Public Health, USA; c Department of Medical Statistics, London School of Hygiene & Tropical Medicine, United Kingdom

**Keywords:** Suicide, Gun violence, G-computation, Alcohol outlets, Firearm dealers

## Abstract

Firearm availability has been linked to firearm self-harm, but the joint relationship with alcohol availability, while supported by theory, has not been examined. This study sought to quantify the separate and joint relations of community firearm and alcohol availability with individual-level risk of (fatal and nonfatal) firearm self-harm. We conducted a case-control study of California residents, 2005–2015, using statewide mortality, hospital, firearm transfer, and alcohol license data. We estimated monthly marginal risk differences per 100,000 in the overall population and in white men aged 50+ under various hypothetical changes to firearm and alcohol availability and assessed additive interactions using case-control-weighted g-computation. In the overall population, non-pawn shop firearm dealer density was associated with firearm self-harm (RD: 0.02, 95% CI: 0.003, 0.04) but pawn shop firearm dealer and alcohol outlet densities were not. Secondary analyses revealed a relationship between firearm sales density and firearm self-harm (RD: 0.07, 95% CI: 0.04, 0.10). There were no additive interactions between measures of firearm and alcohol availability. Among older white men, generally the same exposures were related to self-harm as in the overall population, but point estimates were substantially larger. Findings suggest community-level approaches to reducing firearm sales may help mitigate suicide risk.

## 1. Introduction

Firearms are the most commonly used and deadliest method of suicide in the United States (Conner et al., 2019). Firearm suicide is the 10th leading cause of death and the 4th leading cause of death by injury (WISQARS, 2020). The age-adjusted rate of firearm suicide increased by 25% from 2006 to 2020, affecting all racial/ethnic groups (WISQARS, 2020). However, risk is concentrated among older white men who suffer both the highest rate and greatest number of deaths from this cause (Wintemute, 2015; Pear et al., 2018).

Firearm ownership, also concentrated among older white men (Azrael et al., 2017; Kravitz-Wirtz et al., 2020), is a major risk factor for firearm suicide (Studdert et al., 2020). Suicide attempts are often impulsive acts (Deisenhammer et al., 2009) that are made more deadly when lethal means, such as firearms, are readily available (Conner et al., 2019). Public health interventions targeting firearm ownership directly can be politically challenging, so it is important to identify alternatives, such as community-level interventions that can create a safer environment for at-risk individuals.

Firearm dealers are the primary source of new firearms in a community. Thus, it is plausible that areas with higher firearm dealer density or firearm sales density would have elevated rates of firearm suicide. However, only a few studies have evaluated this relationship and findings are mixed (Chao et al., 2019; Price et al., 2004; Steelesmith et al., 2019; Matthay et al., 2021). Previous research comprised ecological, cross-sectional, and often state-level studies that primarily used Federal Firearm Licenses (FFLs) to measure firearm dealers, which do not account for sales and can be imprecise with regard to timing of dealer openings and closings. As firearm dealers are modifiable features of the local environment, it is particularly important to understand their role in suicide.

Alcohol use is another major risk factor for firearm suicide: an estimated 35% of firearm suicide victims in the United States consumed alcohol shortly before death (Branas et al., 2016). Alcohol outlet density, as a modifiable measure of community-level alcohol availability and norms, provides another opportunity for intervention. Alcohol outlet density has been linked to local drinking behavior, including heavy and binge drinking (Martins et al., 2020; Truong and Sturm, 2007), which, in turn, increases risk for suicide (Sher, 2006). Outlet density has also previously been associated with suicide by any means (Markowitz et al., 2003; Johnson et al., 2009). However, the single study that examined the relationship between alcohol outlet density and firearm suicide, in particular, did not find an association (Branas et al., 2011).

The joint effects of alcohol outlet density and firearm dealer density on firearm self-harm are unknown, but it is plausible that risk is highest in areas where both alcohol and firearms are easily accessible. In this study of California residents, we aim to 1) quantify the association between active firearm dealers and (fatal and nonfatal) firearm self-harm; 2) quantify the association between alcohol outlet density and firearm self-harm; and 3) quantify the joint association between both firearm and alcohol availability and firearm self-harm, testing for additive interaction between the exposures. We additionally examine these associations among older white men, the subset of the population at highest risk for firearm suicide, and explore firearm availability vis-à-vis firearm sales rather than dealers.

## 2. Methods

### 2.1. Study design

We conducted a density-sampled case-control study of California residents from January 2005 through September 2015. Cases were Californians at least 10 years of age who were injured or killed by an intentional self-directed gunshot wound (it is extremely rare for self-directed injuries to be coded as intentional for children under 10). Using American Community Survey data, we selected 4 controls for every case from the general population aged 10 and over, drawing directly from the primary study base (Wacholder et al., 1992a, 1992b). We examined the associations between county-level firearm availability and/or ZIP code tabulation area (ZCTA) level alcohol availability and individual-level risk of fatal and nonfatal self-inflicted firearm injury using case-control-weighted g-computation to estimate risk differences and additive interactions, controlling for individual and community-level confounders (detailed below).

### 2.2. Data and measures

#### Exposures:

The first exposure of interest was community firearm availability. This was measured monthly with Dealer Record of Sale (DROS) data, maintained by the California Department of Justice, from January 2004 through September 2015 (the most recent data available). These data comprise records for nearly every legal handgun transfer in California since 1991 and every long gun transfer since 2014. Thus, DROS data can be used to identify which firearm dealers are actively selling firearms and how many firearms they sell each month. Counties were empirically determined to be the best spatial unit of analysis given the distance typically travelled for firearm acquisition (details in the Web Appendix).

We measured firearm availability as monthly active firearm dealer density per 100,000 residents for each county. We used these to calculate a 12-month moving average to increase stability. “Active” firearm dealers were defined as dealers with at least 1 sale in a given month. Pawn shop and non-pawn dealer densities were measured separately, as they were hypothesized to have different associations with the outcome (only pawn shops have been linked to firearm suicide) (Chao et al., 2019). DROS data do not distinguish pawn shops from other dealers, so we classified dealers as pawn shops if they had “pawn” or “loan” in the business name or email address or if they had a DROS record for redeeming pawned firearms.

The second exposure of interest was community alcohol availability, measured with ZCTA-level alcohol outlet density (the smallest geographic unit in our outcome data), 2004–2015. This is consistent with previous ZIP code-level studies of alcohol outlet density (Johnson et al., 2009; Chen et al., 2009), but avoids misalignment issues that arise when using ZIP codes over time. ZCTAs, created by the Census Bureau, approximate ZIP codes and are stable for 10-year periods. Using annual alcohol outlet data from the California Department of Alcoholic Beverage Control (ABC), we estimated the annual ZCTA density per 100, 000 residents for off-premise outlets (license types 20 and 21) and bars/pubs (license types 23, 40, 42, 48, 61, and 75) separately, as they were previously found to have different associations with violence (Grubesic et al., 2013). We linked the geocoded ABC data to the 2010 ZCTA map to get the number of outlets in each ZCTA-year. To estimate exposures throughout the year, we calculated a moving weighted average of the current and prior year’s density (e.g., the density for April 2005 was calculated as $\frac{4}{12}\;(2005\;\text{density})+\frac{8}{12}\;(2004\;\text{density})$).

#### Cases:

Cases were all individuals in California with a fatal or nonfatal self-inflicted firearm injury (hereafter “firearm self-harm”) from January 2005 through September 2015. Fatal case data were from the California Department of Public Health’s Comprehensive Death Files and nonfatal case data were from the California Department of Health Care Access and Information’s (HCAI’s) emergency department and hospital discharge records. The death data used the International Statistical Classification of Diseases and Related Health Problems, 10th edition (ICD-10) and the injury data used the 9th edition (ICD-9); firearm self-harm was identified with ICD-10 codes X72-74 and with ICD-9 codes E955.0-E955.4 (see Web Table 1 for details). We restricted cases to California residents (for whom we have exposure data) and whenever possible (in 5 cases), we used linked individual-level HCAI records to fill in missing demographic characteristics (e.g., race, ZIP code).

#### Controls:

To sample controls from the residential population in California, we first estimated the annual ZCTA-county population by age, sex, and race using a crosswalk linking ZCTAs to counties; ZCTA age-sex-race subpopulations were allocated to each ZCTA-county unit based on the proportion of the total ZCTA population within the county. Those under 10 years of age were excluded to reflect the study base of our cases. We estimated monthly values by linearly interpolating changes in subgroup population counts and refined the at-risk population estimates by removing cases from the population corresponding to the case’s ZCTA, age, sex, and race. We then calculated the probability of being in each ZCTA-county age-sex-race group at each year-month and randomly sampled controls from this multinomial distribution for every study month at a 4:1 ratio with incident cases.

#### Covariates:

We used theory and previous literature to determine covariates *a priori* using a directed acyclic graph to visualize their relationships (Web Fig. 1). We controlled for the following confounders: individual age, race/ethnicity, sex; ZCTA percent aged 55 and older, percent non-Hispanic white, percent male, urbanicity, median household income z-score (hereafter “income”), percent aged 25 years and older with at least a bachelor’s degree (hereafter “education), and un-employment rate in the civilian work force over age 16 (hereafter “un-employment”). We included spatial lags of each exposure to better isolate within-unit associations. These were calculated as the mean of a given exposure in all adjacent counties or ZCTAs, as appropriate, using queen contiguity weights. We also controlled for year to account for secular trends in firearm self-harm and an indicator for cooler (September–February) vs. warmer (March–August) months, based on the observed seasonal variation in firearm self-harm (Web Fig 2). Finally, we included an indicator for when DROS started recording long gun transactions in 2014. Long gun data were excluded in sensitivity analyses (detailed below). Additional details on data sources and definitions are provided in the Web Appendix.

### 2.3. Statistical analysis

To estimate risk differences and additive interactions, we used case-control-weighted (CCW) g-computation. G-computation is a parametric substitution estimator, i.e., the outcomes are estimated under specified hypothetical changes to the exposure(s), which are substituted into the parametric model. The case-control sampling design is accommodated by reweighting: cases are weighted with the population prevalence of the outcome and controls are weighted with (1-prevalence)/(control: case ratio) (van der Laan and Rose, 2011; Pearl et al., 2016). See the Web Appendix for additional details.

The CCW g-computation estimates were based on multi-level models that allowed us to estimate associations between ecological exposures and individual risks while controlling for individual-level confounders (Greenland, 2001). Specifically, we used CCW logistic regression models with cluster-robust standard errors to account for non-independence of observations from individuals being nested within counties, ZCTAs, and year-months. We included multiplicative interactions between the firearm and alcohol availability variables and retained those with p < 0.20 (Jewell, 2004). We created scatterplots with smoothed lines of fit to visualize nonlinearity in the bivariate relationships between each continuous variable and the log-odds of the modeled outcome. To minimize overfitting, we modeled variables with restricted cubic splines only if they appeared nonlinear and they were significantly associated with the outcome (at alpha = 0.10) when modeled with or without a spline.

We estimated 2 parameters for 2 populations: the entire state population 10+ years old and the highest-risk subpopulation, white men 50+ years old. The first parameter is the overall risk difference (RD_overall_) comparing firearm self-harm rates when a given exposure (non-pawn, pawn, off-premise alcohol outlet or bar/pub density) is set to high density with firearm self-harm rates when the exposure is set to low density. “High” and “low” were determined empirically, using the observed highest and lowest values in each county or ZCTA over the study period to avoid positivity violations (Web Table 2). The second parameter estimates what we call the conservative population attributable risk (RD_cPAR_), comparing the outcome under the observed value of a given exposure with a scenario in which the exposure is set to low density. We also calculated the corresponding risk ratios (RR) to provide additional context for understanding the absolute differences presented.

In addition to these individual exposures, we examined the joint exposures of firearm availability and alcohol outlets. We also calculated additive interactions by subtracting the sum of the individual RDs (“expected”) from the joint RD (“observed”). We examined additive interactions to better isolate cases that would only occur in the presence of high firearm and alcohol availability but not in the presence of either alone; such isolation cannot be achieved with multiplicative interaction (VanderWeele and Knol, 2014). Bias corrected and accelerated nonparametric bootstrapped confidence intervals were calculated for all point estimates (n runs = 400). (Carpenter and Bithell, 2000).

### 2.4. Secondary and sensitivity analyses

Secondary analyses measured firearm availability with firearm sales density per 100,000 instead of firearm dealer density, enabling us to determine which of these measures is more relevant to firearm self-harm. To focus on new firearms entering a community, we limited transactions in DROS to sales (excluding pawn redemptions, private party transfers, curio/relics, loans, and non-roster peace officer transfers). To determine whether results were sensitive to the inclusion of long gun data, sensitivity analyses re-estimated the RD_overall_ and RR_overall_ for the full population after removing long guns from the DROS data, thereby excluding long gun sales as well as dealers that only sold long guns.

Analyses were performed with R 4.0.2, Stata/MP 13.1, and ArcGIS 10.7. This study was approved by the California Health and Human Services Agency’s Committee for the Protection for Human Subjects; University of California, Berkeley’s Committee for the Protection for Human Subjects; and the University of California, Davis Institutional Review Board.

## 3. Results

From January 2005 through September 2015, there were 17,277 intentional self-directed firearm injuries in California among residents. One case was removed for being under the age of 10. Records missing ZCTA, age, sex, or race were dropped (n = 103), leaving 17,173 cases remaining. With 68,692 controls, this yielded a sample size of 85,865. We dropped 24 cases and 72 controls with missing community-level covariate information. In all, 0.7% of cases and 0.1% of controls were dropped for missingness. Several continuous variables were highly skewed. To minimize the potential bias from these extreme outliers, we removed all observations with an exposure or covariate value more than 10-times the interquartile range (IQR) below the 1st quartile or greater than the 3rd quartile of the variable’s distribution (n = 500 cases and 910 controls). In visualizing the data, we identified and removed an additional 3 observations with extreme covariate values, yielding a final sample size of 84,356 (16,648 cases and 67,708 controls). Controls closely matched the Californian population from which they were drawn (Web Table 3).

Table 1 presents the individual- and community-level characteristics of study participants. Firearm self-harm cases were more likely than controls to be male (88.0% vs. 49.3%), white (76.5% vs 42.1%), 50+ years old (57.4% vs. 34.2%), and live outside of urban areas (16.1% vs. 7.7%). Alcohol outlet, firearm dealer, and firearm sales densities were all higher among cases than controls.

Table 2 displays the monthly risk per 100,000 of firearm self-harm, weighted to be representative of the state population, by tertile of each exposure. On average, the monthly rate of firearm self-harm was 0.41 injuries per 100,000. The risk of firearm self-harm injury increased from lowest to highest tertile across all exposures. The spatial distribution of the exposures and outcome are displayed in Web Fig. 3.

**Fig. 1.**
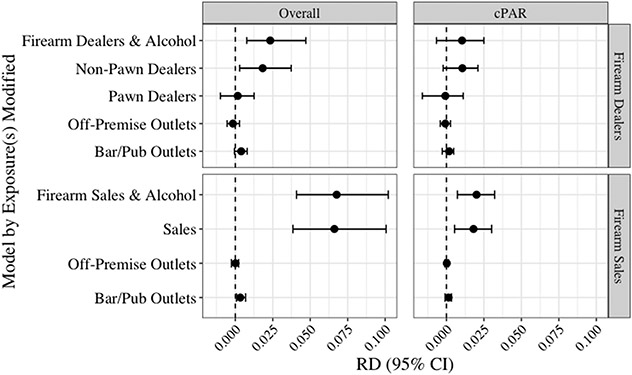
Adjusted Risk Differences for Firearm Self-Harm per 100,000 Residents per Month^a^ a. “Firearm dealers” includes both non-pawn and pawn dealers. “Alcohol” includes both off-premise outlets and bars/pubs. Non-pawn dealers, pawn dealers, and firearm sales density were measured per 100,000 population at the county level. Off-premise outlets and bars/pubs were measured per 100,000 population at the ZCTA level.

Fig. 1 presents the adjusted CCW g-computation results for firearm self-harm in the full population (corresponding RRs are in Web Table 4). Measuring firearm availability with dealers, in single exposure models, only non-pawn firearm dealer density was associated with firearm self-harm and the magnitude of association was modest (RD_overall_: 0.02, 95% CI: 0.003, 0.04). The joint association between firearm dealer and alcohol outlets was nearly identical, as the other individual point estimates were close to zero and there was no additive interaction (Table 3).

In secondary analyses examining firearm sales density, there was a moderately increased monthly risk of 0.07 injuries per 100,000 (95% CI: 0.04, 0.10), representing a 17% relative increase (95% CI: 1.10, 1.26), or about 21 firearm self-harm injuries per month (Fig. 1; Web Table 4). This was essentially unchanged in the joint model as alcohol outlet densities were not meaningfully associated with firearm self-harm and there was no interaction between firearm sales and alcohol outlet density (Table 3).

As expected, the RD_cPAR_ estimates were attenuated compared with the RD_overall_, but they followed a similar pattern (Fig. 1). In the primary analyses, neither the individual nor the joint exposures were associated with firearm self-harm. In secondary analyses using firearm sales to measure firearm availability, the individual alcohol outlet density exposures were null, but firearm sales density was associated with small but significant increases in the risk of firearm self-harm (sales RD_cPar_: 0.02, 95% CI: 0.01, 0.03), equivalent to about 70 fewer firearm self-harm injuries per year (there were 1513 such injuries per year on average during the study period).

**Fig. 2.**
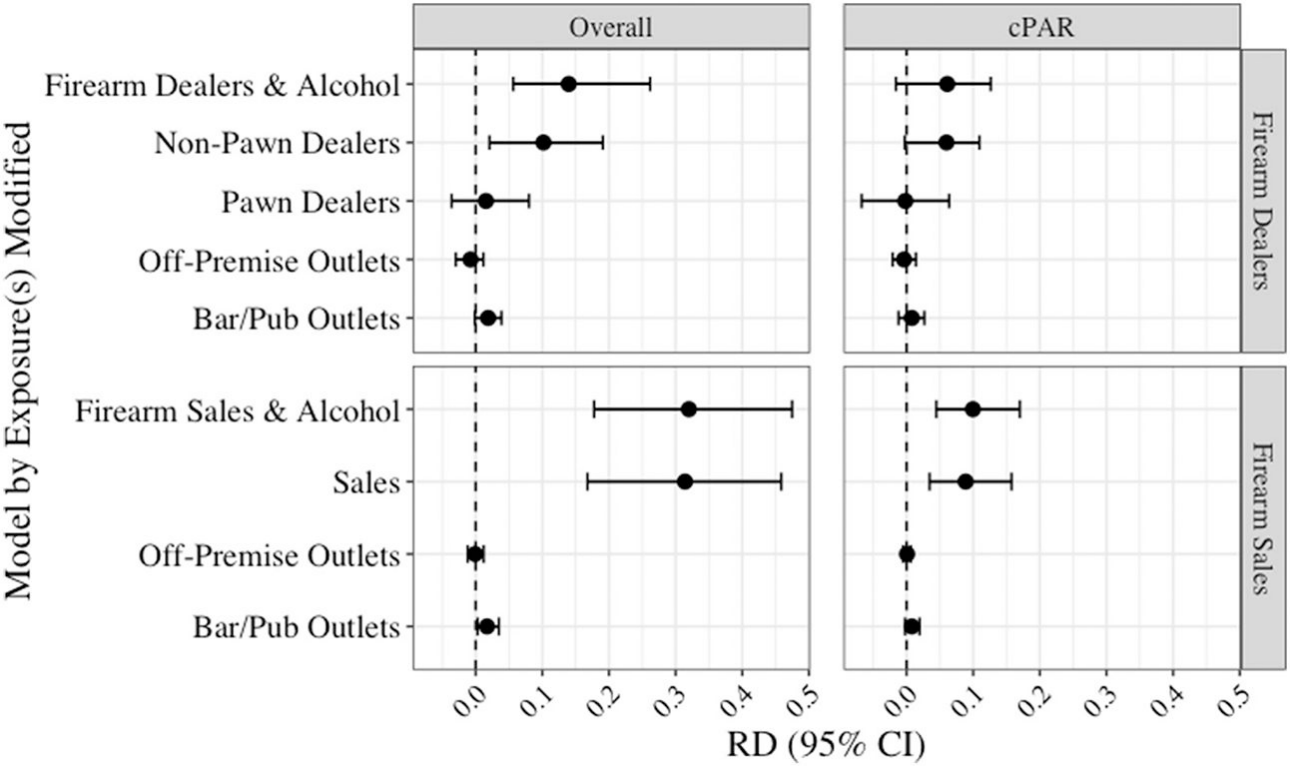
Adjusted Risk Differences for Firearm Self-Harm per 100,000 Residents per Month Among White Men Aged 50+^a^ a. “Firearm dealers” includes both non-pawn and pawn dealers. “Alcohol” includes both off-premise outlets and bars/pubs. Non-pawn dealers, pawn dealers, and firearm sales density were measured per 100,000 population at the county level. Off-premise outlets and bars/pubs were measured per 100,000 population at the ZCTA level.

Fig. 2 presents the corresponding results for the high-risk subgroup analysis of white men aged 50+ (RRs are presented in Web Table 5). The average monthly rate of firearm self-harm in this population was 2.02 injuries per 100,000. Overall, patterns of association were similar with the statewide analysis, but the magnitudes were larger in the high-risk group. In the primary models, only non-pawn firearm dealer density was individually associated with increased risk of firearm self-harm among older white men (RD_overall_ 0.10, 95% CI: 0.02, 0.19). This was also the main contributor to the estimate for the joint association for firearm dealer and alcohol outlet density together (RD_overall_ 0.14, 95% CI: 0.06, 0.26). As in the population-wide analysis, there were no additive interactions between measures of firearm and alcohol availability (Table 3).

In secondary analyses using firearm sales to measure availability, we found sales density to be associated with substantially increased risk of firearm self-harm among the high-risk population (RD_overall_: 0.31, 95% CI: 0.17, 0.46; Fig. 2), corresponding to a 17% relative increase (95% CI: 1.09, 1.25; Web Table 5). Bar/pub density was also associated with slightly increased risk (RD_overall_ 0.02, 95% CI: 0.003, 0.03). Together, high densities of firearm sales and alcohol outlets were associated with an additional monthly risk of 0.32 injuries per 100,000 (95% CI: 0.18, 0.47) among older white men, due almost entirely to firearm sales. There was no evidence of interaction (Table 3).

Among the high-risk population, the RD_cPAR_ was null for all individual and joint exposure models using firearm dealer density to measure firearm availability (Fig. 2). In secondary analyses, we found that the observed firearm sales density compared with low sales density was associated with an increased monthly risk of 0.09 injuries per 100,000 among older white men (95% CI: 0.03, 0.16). Together, the joint association of firearm sales and alcohol availability had nearly the same point estimate as firearm sales alone (RD_cPAR_: 0.10, 95% CI: 0.04, 0.17).

Results from the sensitivity analyses removing long guns from the DROS data are displayed in Web Table 6. This reduced the magnitude of our findings for the RD_overall_ in the statewide population by about half in all firearm-related exposure models (single exposure alcohol outlet density models were unaffected). Non-pawn firearm dealers were no longer associated with firearm self-harm. Firearm sales density remained the community feature most strongly associated with increased risk of firearm self-harm (RD_overall_: 0.03, 95% CI: 0.02, 0.05).

## 4. Discussion

We found that non-pawn shop firearm dealer density was associated with firearm self-harm, but not pawn dealer density. Across all models, alcohol outlet density was weakly or not at all associated with firearm self-harm and there were no additive interactions between measures of firearm and alcohol availability. Secondary analyses revealed a moderate relationship between firearm sales density and firearm self-harm, with risk 17% higher if all communities had high versus low sales density, corresponding to about 257 additional firearm self-harm injuries per year. Firearm sales density also had a meaningful magnitude of association with firearm self-harm risk in RD_cPAR_ models, suggesting that, to the extent these findings reflect a causal relationship, an intervention to reduce firearms sales density could have a meaningful impact on firearm self-harm—particularly among those at highest risk, for whom associations were substantially larger than those for the statewide population across all exposures of interest.

Few studies have previously evaluated the association between firearm dealer density and firearm suicide. One county-level study found firearm dealer density to be associated with increased firearm suicide rates (Steelesmith et al., 2019), and a state-level study found pawn shop density, but not non-pawn density, to be associated with increased suicide rates (Chao et al., 2019). However, a separate state-level study with better control of confounding than its counterpart found no association (Price et al., 2004). None of these studies are directly comparable to ours, since we estimated the individual-level risk of firearm self-harm with a multi-level design while the others used ecological designs to examine changes in group-level rates of firearm suicide. We also examined different populations and controlled for different sets of confounders. In general, the findings of these past ecologic studies were not mirrored in this multi-level analysis, as we found a modest association between non-pawn firearm dealers and risk of self-harm in the statewide and high-risk group analyses (however, this association was not significant in the sensitivity analysis excluding long guns).

Associations were much stronger when we measured firearm availability with firearm sales rather than dealers in both the general and high-risk populations. To our knowledge, ours is the first study to evaluate the association between community-level sales and individual-level risk of firearm self-harm. At the individual level, it is well established that purchasing a handgun is associated with extremely high relative risk of firearm suicide (Studdert et al., 2020). This risk extends to others living with the firearm owner, including children (Swanson et al., 2020; Anglemyer et al., 2014). Although about 70% of firearm suicides are from handguns (Wintemute et al., 1988; Nestadt et al., 2020), findings from our sensitivity analyses suggest that interventions targeting only handguns would have more limited benefits. However, the differences between high and low densities of firearm dealers and sales were smaller in the sensitivity analyses excluding long guns, which may partially explain why the associations were attenuated. Future studies should use additional years of long gun data to better estimate the relationship between long gun sales and firearm self-harm. Currently, firearm transaction records are only available for the State of California. Our findings illustrate the public health value in allowing researchers access to such data, as sales (and ownership) data are essential to studying the role of community- and individual-level firearm accessibility in firearm violence.

Taken together, our findings indicate that firearm dealers may play a role in firearm suicide through increasing the accessibility of firearms to at-risk individuals, primarily through sales. Community-level interventions could consider targeting local volume of firearm sales. Private companies can aid in this effort by ending or limiting sales of firearms. This has worked previously: Walmart’s decision to stop selling handguns in 1994 was associated with a 3.3–7.5% reduction in the firearm suicide rate in affected counties (Ayres et al., 2020). More recently, Dick’s Sporting Goods stopped selling assault rifles after the mass shooting in Parkland, Florida and has since been scaling back the number of stores selling firearms of any kind. Based on the Walmart findings, other “big-box” stores selling firearms should consider following suit. Firearm dealers have been willing to participate in other suicide prevention efforts, such as providing temporary storage of firearms during periods of heightened risk (Runyan et al., 2017; Tung et al., 2019) and displaying educational material about suicide and firearm safety (Vriniotis et al., 2015). Such efforts, if successful, may reduce the risk associated with community-level firearm availability.

With respect to alcohol outlet density, the weak and null associations in the full population analyses are consistent with a previous case-control study of Philadelphia residents (Branas et al., 2011). Among those at highest risk, we found that a high density of bars/pubs was associated with a slightly increased risk of firearm self-harm (corresponding to a 1% relative increase). Similarly, a ZIP code-level ecological study of suicide and suicide attempts by any means in California found bar density to be a risk factor (Johnson et al., 2009). The specific role of bars in suicide deserves further exploration. Overall, there is little evidence that community-level interventions to reduce alcohol outlet density would substantially reduce risk of firearm suicide. Nevertheless, previous research indicates that certain subsets of the population may benefit and there may be other public health considerations supporting such an intervention (Markowitz et al., 2003; Campbell et al., 2009).

## 5. Limitations

This study’s findings should be considered in light of its limitations. The outcome could be subject to misclassification, as it can be challenging to distinguish between unintentional deaths and suicides, but we would not expect this to be differential by firearm dealer or alcohol outlet exposure levels. Furthermore, this is less of a concern with firearm deaths than certain other forms of suicide (e.g., drug overdose). Another limitation is that comparison between high and low density was sometimes quite small, particularly for pawn density, because we did not extrapolate beyond the observed highest and lowest value for each community. Our estimates, therefore, reflect both the adjusted association of interest and the degree of underlying variation in the exposure variables. Furthermore, we had a limited set of individual-level covariates, so there may have been residual confounding. We cannot be sure that our results would generalize outside of California, and such generalizability would be complicated by California’s stringent firearm laws, which could modify the associations of interest.

## 6. Conclusions

Risk factors for firearm self-harm come from multiple social-ecological levels, including the social and physical environments in which we live. Successful suicide prevention strategies will therefore include individual-level interventions, such as gun violence restraining orders (Swanson et al., 2017, 2019), as well as broad measures that reduce risk in the environment. Our findings suggest that community-level approaches targeting pawn shop firearm dealer density or alcohol outlet density are unlikely to reduce firearm suicide, but that reducing non-pawn firearm dealer and especially firearm sales density may help, particularly for those at greatest risk.

## Supplementary Material

Supplementary Material

## Figures and Tables

**Table 1:** Characteristics of cases and controls.

	Cases^a^	Controls
Total	16,648	67,708
**Individual Characteristics**		
Sex, N (%)		
Male	14,654 (88.02)	33,389 (49.31)
Race/ethnicity, N (%)		
Non-Hispanic white	12,738 (76.51)	28,535 (42.14)
Non-Hispanic Black	674 (4.05)	4005 (5.92)
Hispanic	2249 (13.51)	23,944 (35.36)
Asian	689 (4.14)	9578 (14.15)
Native American	55 (0.33)	289 (0.43)
Multiracial	243 (1.46)	1357 (2.00)
Age Group, N (%)		
10-19	629 (3.78)	11,079 (16.36)
20-29	2035 (12.22)	11,480 (16.96)
30-39	1915 (11.50)	10,951 (16.17)
40-49	2514 (15.10)	11,046 (16.31)
50-59	3430 (20.60)	10,081 (14.89)
60-69	2493 (14.97)	6681 (9.87)
70-79	1881 (11.30)	3847 (5.68)
80+	1751 (10.52)	2543 (3.76)
**ZCTA-Level Characteristics**		
Alcohol Outlet Density per 100,000 residents, Median (25th, 75th pctl)		
Off-premise	70.53 (52.83, 94.06)	65.79 (49.98, 85.54)
Bar/pub	11.89 (6.04, 21.21)	10.50 (5.38, 17.54)
Urbanicity, N (%)		
Urban	13,965 (83.88)	62,518 (92.33)
Suburban	1594 (9.57)	3587 (5.30)
Rural	1089 (6.54)	1603 (2.37)
Demographics, Median (25th, 75th pctl)		
% Male	49.32 (48.55, 50.15)	49.28 (48.53, 50.12)
% Non-Hispanic white	44.10 (21.60, 65.09)	36.82 (17.55, 57.65)
% Age 55+	21.53 (17.29, 26.65)	23.36 (19.00, 28.64)
Median household income	60,530 (47,348, 79,441)	62,374 (48,350, 84,109)
% Bachelor’s degree+	26.07 (15.67, 40.33)	27.25 (15.93, 41.37)
% Unemployed	9.45 (7.45, 11.99)	8.26 (6.48, 10.73)
**County-Level Characteristics**		
Firearm Dealer or Sales Density per 100,000 residents, Median (25th, 75th pctl)		
Non-pawn dealer	0.87 (0.45, 1.52)	0.74 (0.41, 1.17)
Pawn dealer	0.90 (0.60, 1.57)	0.79 (0.49, 1.15)
Firearm sales	37.61 (24.11, 66.18)	34.27 (22.56, 59.92)

a 70 individuals have multiple self-directed firearm injuries.

**Table 2:** Risk of firearm self-harm per 100,000 residents per month by tertile of firearm dealer, firearm sales, and alcohol outlet density.

	Tertile^a^	Firearm Self-Harm^b^
12-month Firearm Dealer or Sales Density per 100,000 residents		
Non-pawn dealers	1	0.30
	2	0.40
	3	0.54
Pawn dealers	1	0.29
	2	0.40
	3	0.56
Firearm sales	1	0.33
	2	0.41
	3	0.49
12-month Alcohol Outlet Density per 100,000 residents		
Off-premise outlets	1	0.35
	2	0.38
	3	0.50
Bar/pub outlets	1	0.36
	2	0.38
	3	0.49

a 1 is the lowest tertile and 3 is the highest.

b Estimates are weighted to be representative of the population.

**Table 3:** Additive interaction estimates.

Study Population	Interactions Tested^a^	RD Overall Interaction (95% CI)
Total population	Firearm dealers & alcohol outlets	0.001 (−0.003, 0.013)
	Firearm sales & alcohol outlets	−0.002 (−0.003, 0.000)
White men aged 50+	Firearm dealers & alcohol outlets	0.012 (−0.009, 0.128)
	Firearm sales & alcohol outlets	−0.010 (−0.018, 0.002)

a “Firearm dealers” includes both non-pawn and pawn dealers. “Alcohol outlets” include both off-premise outlets and bars/pubs.
