# Supplementary material for: The role of firearm and alcohol availability in firearm suicide: A population-based weighted case-control study
Source: Health Place. Author manuscript; Available in PMC 2023 May 2. (PMC10153632; doi:10.1016/j.healthplace.2023.102969)
Supplement: Supplementary Material [file NIHMS1892197-supplement-Supplementary_Material.pdf]

Supplement: The Role of Firearm and Alcohol Availability in Firearm Suicide: A Population-Based Weighted Case-Control Study

Veronica A. Pear, Garen J. Wintemute, Nicholas P. Jewell, Jennifer Ahern

Web Appendix

Spatial unit of firearm density

To determine the best spatial unit of analysis, we geocoded paired dealer and purchaser addresses in the Dealer Record of Sale data to determine the travel distance between firearm dealers and purchasers. As 70% of purchasers and dealers were in the same county, we chose this as the unit of analysis (worse-performing administrative boundaries were Public Use Microdata Areas, cities, and ZIP codes).

Covariate details

Individual demographic variables were taken directly from the hospital and death data. Race/ethnicity included the following categories: non-Hispanic white, non-Hispanic Black, Hispanic, Asian, Native American, and multiracial. ZCTA income, education, and unemployment data were from the American Community Survey 5-year estimates for 2007-2011 and 2012-2016.[1] We used 2010 census data to calculate the percent of the population in each ZCTA that was in an urban area (>=50,000 people), an urban cluster (2,500-49,999 people), or a rural area (<2,500 people) and then defined each ZCTA as urban, suburban, or rural based on the category with a plurality. ZCTA demographic data (age, sex, race) were calculated monthly using the interpolated population data describe in the methods, transformed into 12 month rolling averages.

Case-Control-Weighted G-Computation

The CCW g-computation formula is as follows:

$$\Psi\,(P) = E_w\{[\,I(Y_{i=1}) * q(t)[E(Y \mid A_1{=}1, A_2{=}1, W_i)]\,] + [\,I(Y_{i=0}) * \frac{\bar{q}(t)}{J(t)}[E(Y \mid A_1{=}1, A_2{=}1, W_i)]\,]\} \,/$$

$$E_w[\,I(Y_{i=1})q(t) + I(Y_{i=0})\frac{\bar{q}(t)}{J(t)}\,] \; -$$

$$E_w\{[\,I(Y_{i=1}) * q(t)[E(Y \mid A_1{=}0, A_2{=}0, W_i)]\,] + [\,I(Y_{i=0}) * \frac{\bar{q}(t)}{J(t)}[E(Y \mid A_1{=}0, A_2{=}0, W_i)]\,]\} \,/$$

$$E_w[\,I(Y_{i=1})q(t) + I(Y_{i=0})\frac{\bar{q}(t)}{J(t)}\,],$$

where Ψ is the risk difference at the true data distribution (P); q_0 and $\bar{q}_0$ are the case and control weights, respectively, at each year-month (t); $E(Y \mid A_1{=}1, A_2{=}1, W{=}w)$ is the expected value of the outcome (Y) when both exposures (A_1 and A_2) are set to a given value, such as 1, adjusting for confounders (W); and E_w is the expectation over W.

References

1. ACS 2011 & 2016 (5-year estimates). US Census Bureau, Social Explorer. 2020. www.socialexplorer.com

Web Table 1: ICD-9 and ICD-10 Codes Used to Identify Firearm Self-Harm

**Description**	**ICD-9 Code**	**ICD-10 Code**
Suicide & self-inflicted injury by:		
Handgun	E955.0	X72
Shotgun	E955.1	X73
Hunting rifle	E955.2	X73
Military firearms	E955.3	X73
Other and unspecified firearm	E955.4	X74

Web Figure 1: Directed Acyclic Graph

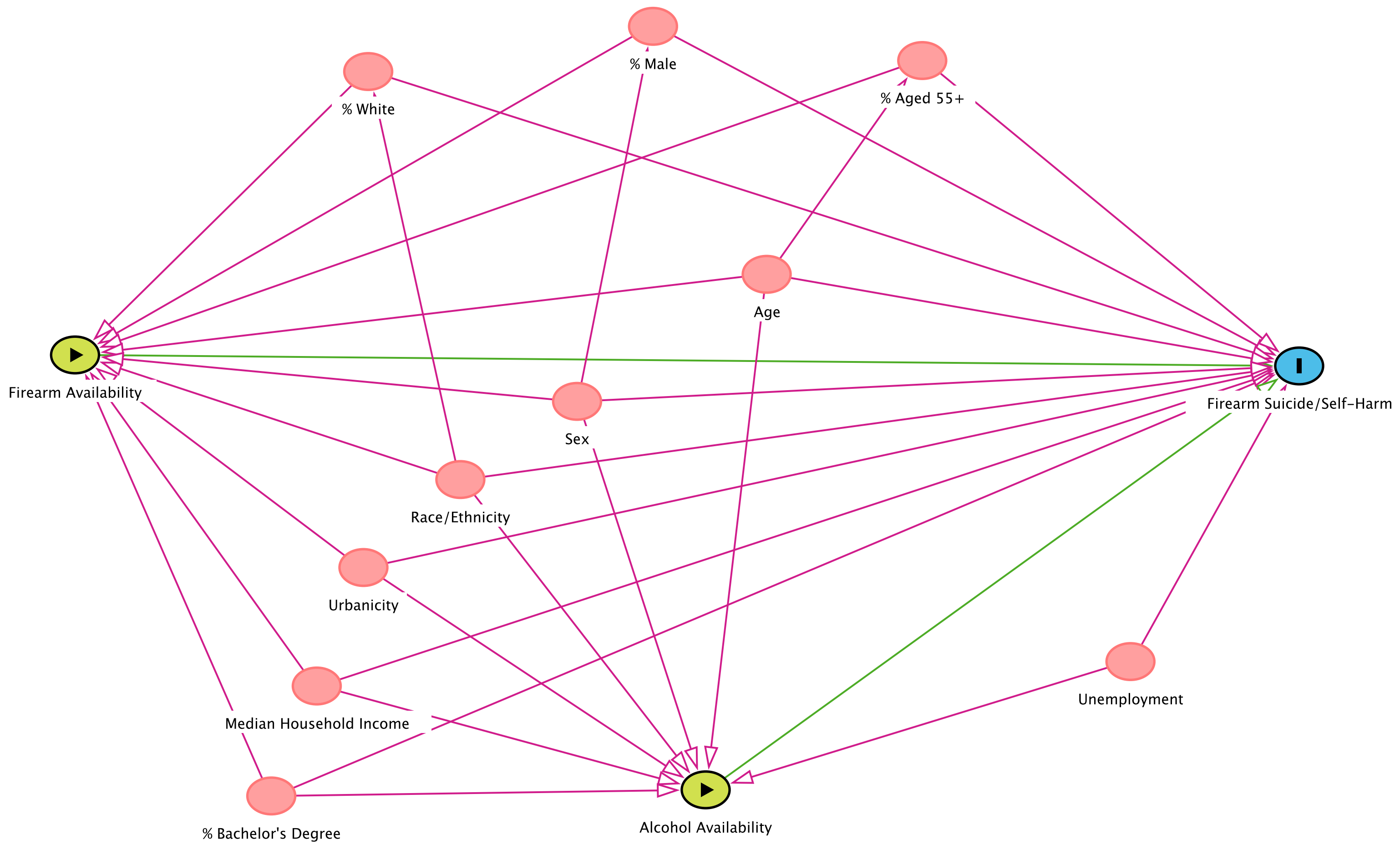

Web Figure 2: Monthly Firearm Self-Harm Injuries

A. Timeseries

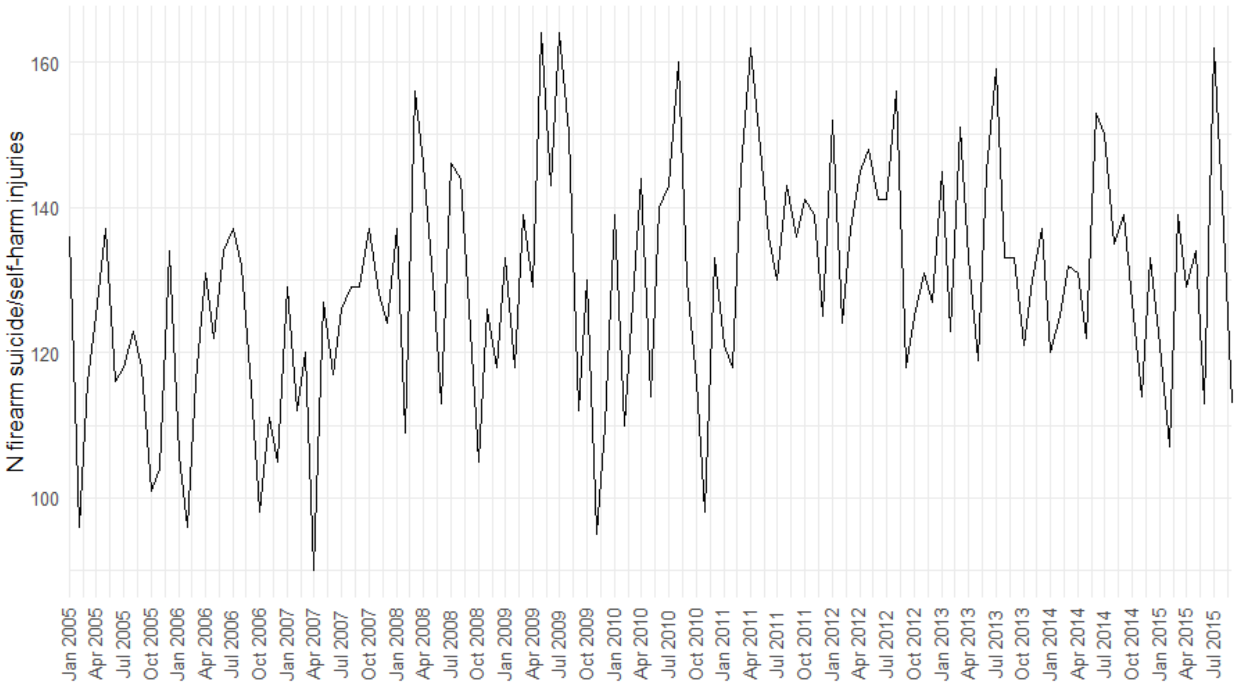

B. Monthly Variation

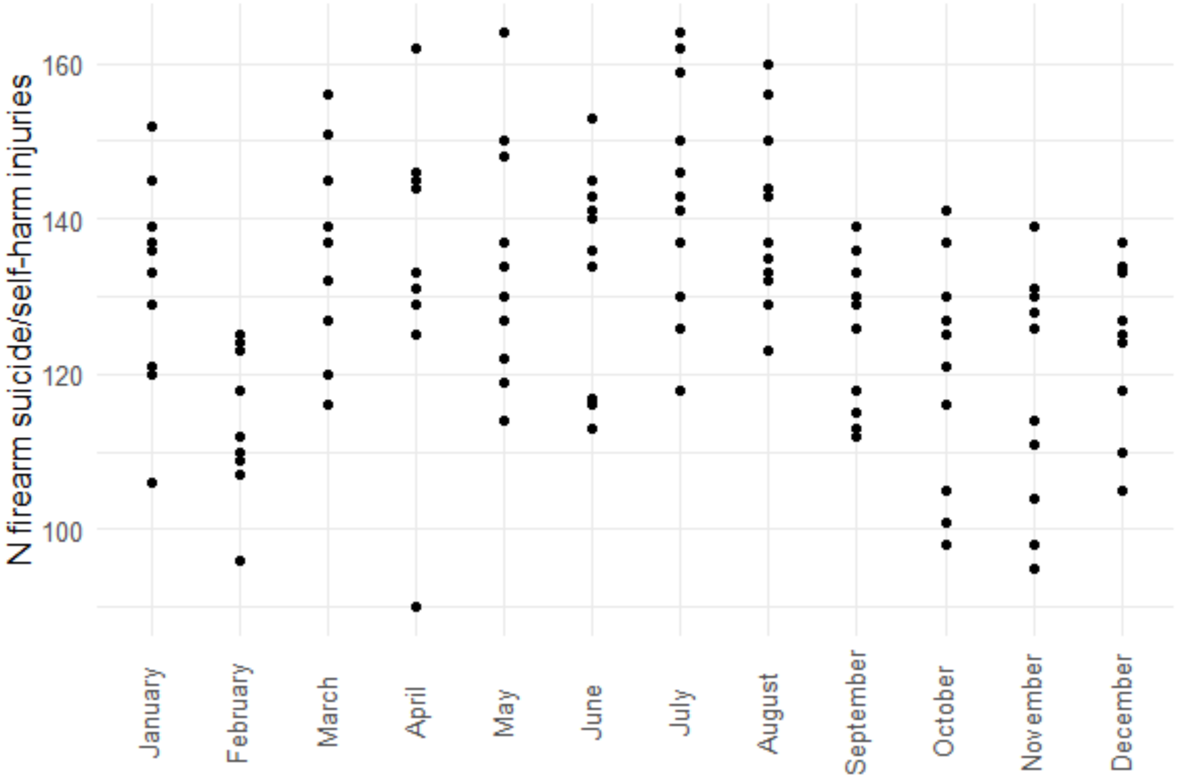

Web Table 2: Range of Firearm and Alcohol Availability Measures[a]

Exposure	Mean Highest Density	Mean Lowest Density	Mean Difference
County-level measures			
Non-pawn dealers	4.95	1.59	3.36
Pawn dealers	3.59	1.61	1.98
Firearm sales	164.33	21.55	142.78
ZCTA-level measures			
Off-premise outlets	107.82	85.23	22.58
Bars/Pubs	23.70	14.60	9.10

a. All densities are measured per 100,000 population.

Web Table 3: Demographic Characteristics of Controls and Residents of California

	Self-Harm Controls[a]	California State > 9 Yrs, 2010
Total	67,708	32,216,784
Sex, N (%)		
Male	33,389 (49.31)	15,944,211 (49.49)
Race/Ethnicity, N (%)		
Non-Hispanic white	28,535 (42.14)	13,694,942 (42.51)
Non-Hispanic Black	4,005 (5.92)	1,919,614 (5.96)
Hispanic	23,944 (35.36)	11,370,655 (35.29)
Other	11,224 (16.58)	5,231,573 (16.24)
Age Group, N (%)		
10-19	11,079 (16.36)	5,414,870 (16.81)
20-29	11,480 (16.96)	5,510,358 (17.10)
30-39	10,951 (16.17)	5,147,047 (15.98)
40-49	11,046 (16.31)	5,298,950 (16.45)
50-59	10,081 (14.89)	4,766,848 (14.80)
60-69	6,681 (9.87)	3,135,755 (9.73)
70-79	3,847 (5.68)	1,738,749 (5.40)
80+	2,543 (3.76)	1,204,207 (3.74)

a. 70 individuals have multiple self-directed firearm injuries. Controls are those used in analysis, after making exclusions due to missingness and extreme outlying values.

Web Figure 3. Spatial Distribution of Exposures and Outcome, in Deciles[a]

A. Firearm Self-Harm Injury Rate

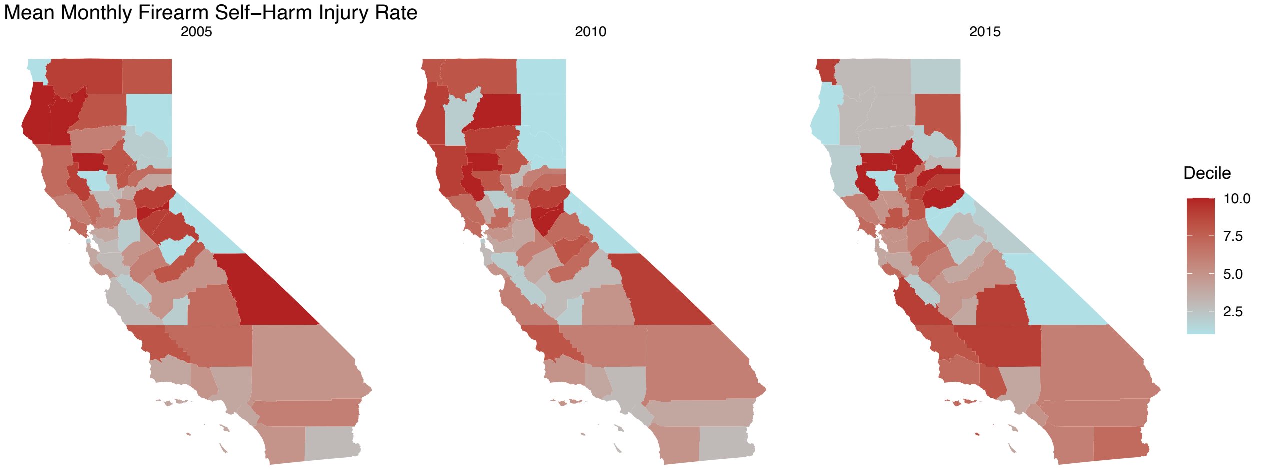

B. Non-Pawn Firearm Dealer Density

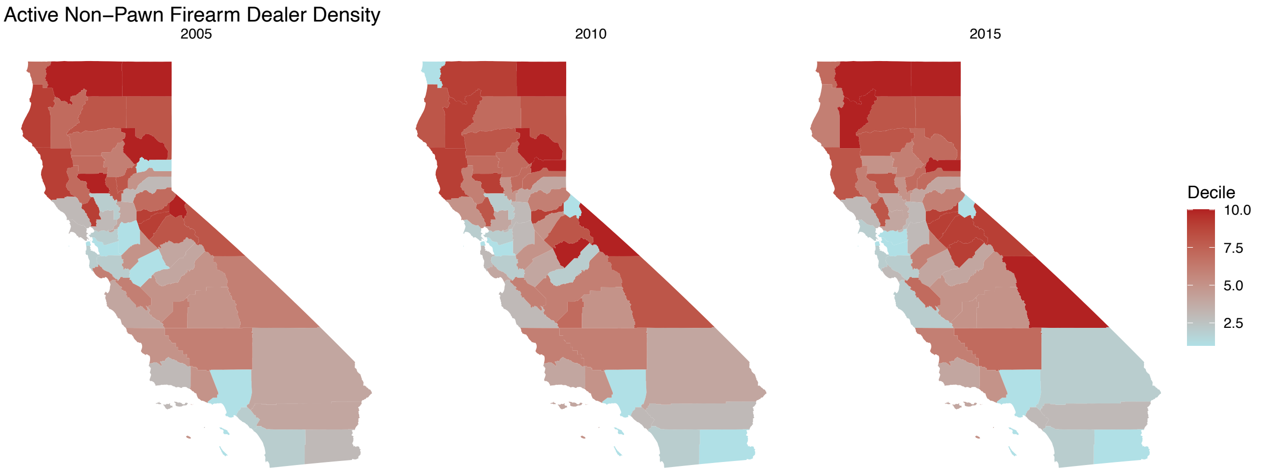

C. Pawn Firearm Dealer Density

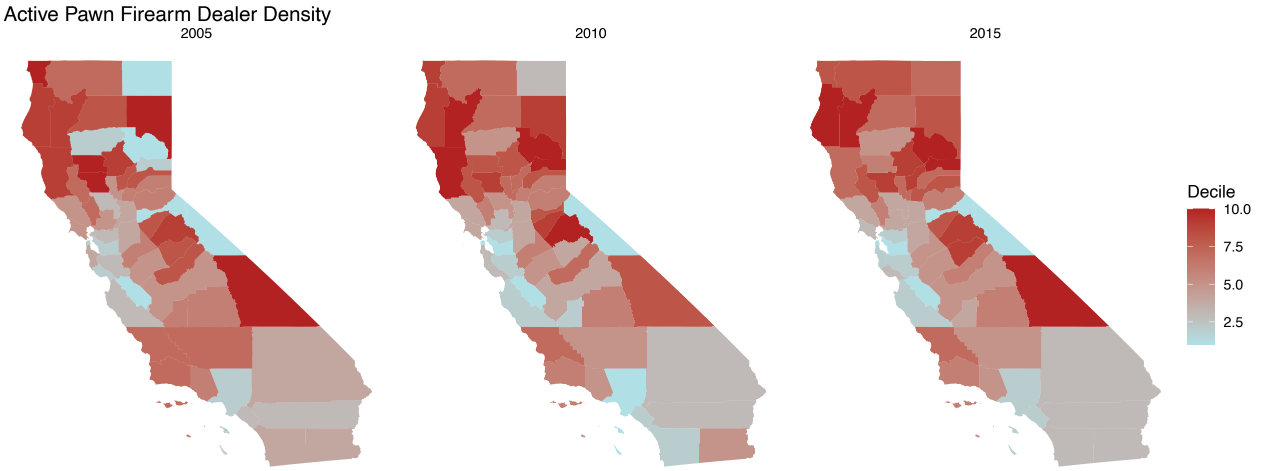

D. Firearm Sales Density

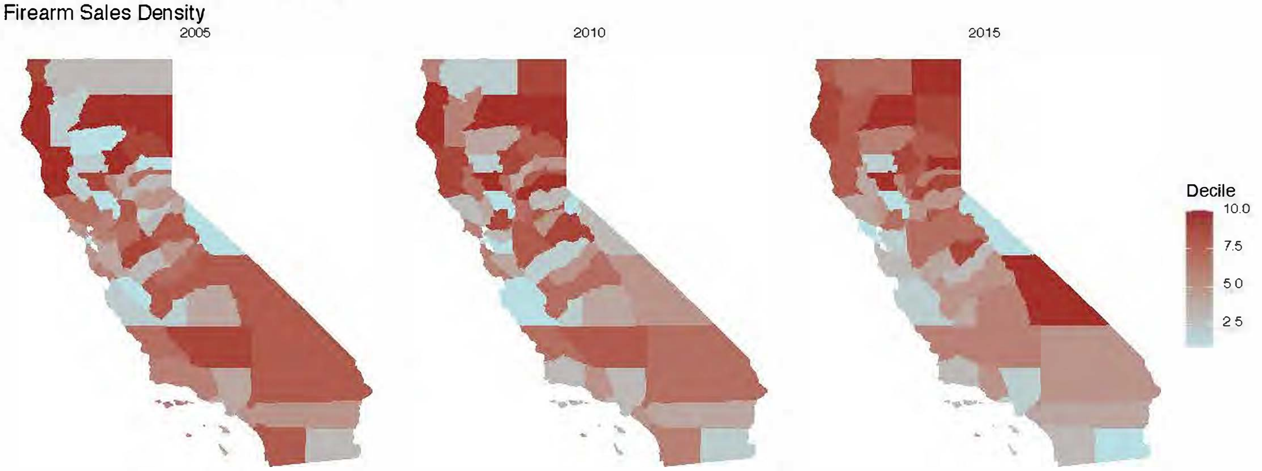

E. Off-Premise Alcohol Outlet Density

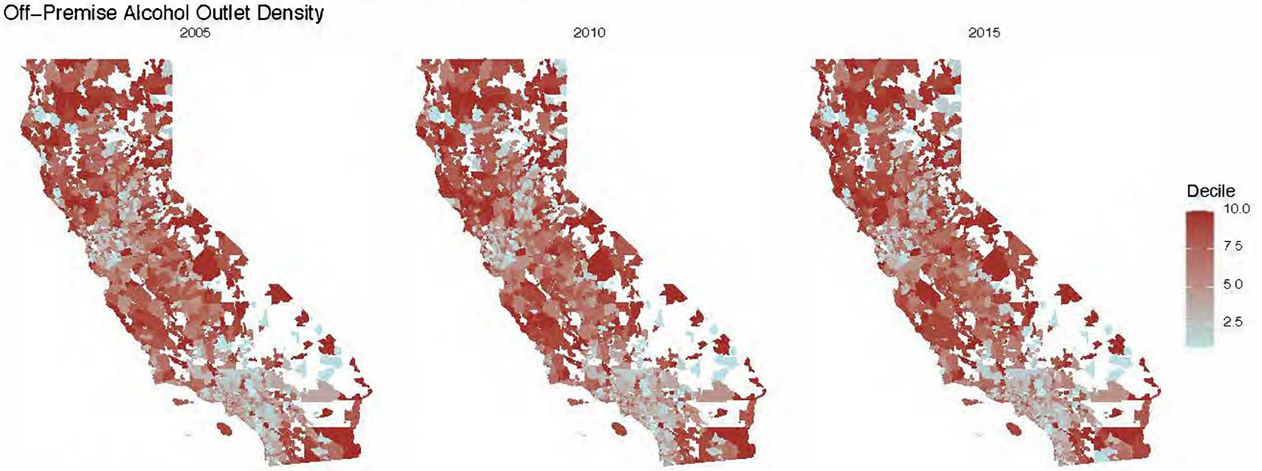

F. Bar/Pub Density

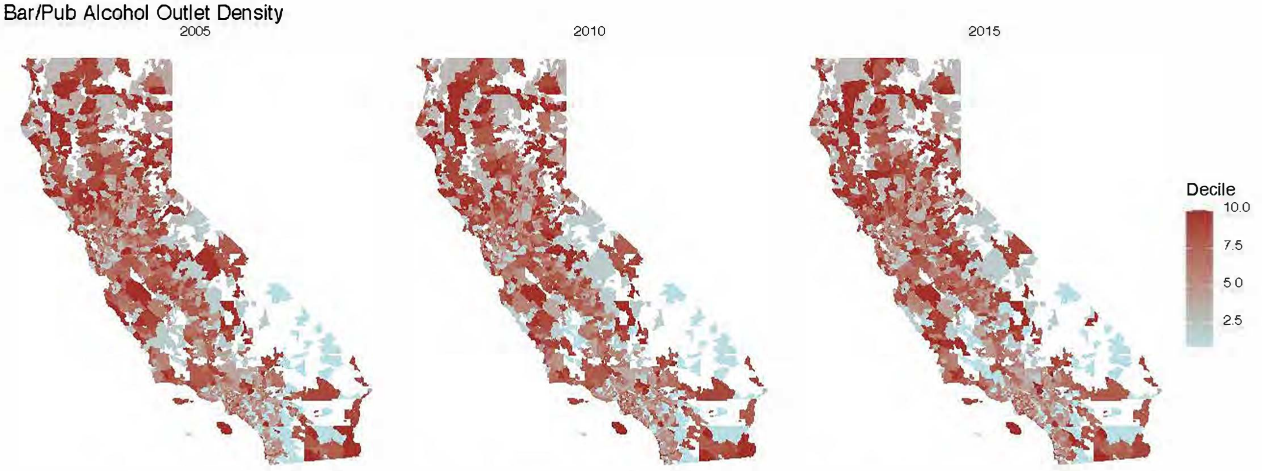

a. Monthly county/ZCTA rates were averaged for 2005, 2010, and October 2014-September 2015 (the last 12 months of the study). Deciles were calculated for each of these 12-month periods individually.

Web Table 4: Adjusted Relative Risks of Firearm Self-Harm

Measure of Firearm Availability	Model by Exposure(s) Adjusted[a]	RR Overall (95% CI)	RR cPAR (95% CI)
Firearm Dealers	Dealers & Alcohol	1.06 (1.02, 1.12)	1.03 (0.98, 1.07)
	Non-Pawn	1.05 (1.01, 1.10)	1.03 (0.99, 1.05)
	Pawn	1.00 (0.98, 1.03)	1.00 (0.96, 1.03)
	Off-Premise	1.00 (0.99, 1.01)	1.00 (0.99, 1.01)
	Bars/Pubs	1.01 (1.00, 1.02)	1.00 (0.99, 1.01)
Firearm Sales	Sales & Alcohol	1.17 (1.10, 1.27)	1.05 (1.02, 1.09)
	Sales	1.17 (1.10, 1.26)	1.05 (1.01, 1.08)
	Off-Premise	1.00 (0.99, 1.01)	1.00 (1.00, 1.00)
	Bars/Pubs	1.01 (1.00, 1.02)	1.00 (1.00, 1.01)

a. "Dealers" includes both non-pawn and pawn dealers. "Alcohol" includes both off-premise outlets and bars/pubs. Non-pawn dealers, pawn dealers, and firearm sales density were measured per 100,000 population at the county level. Off-premise outlets and bars/pubs were measured per 100,000 population at the ZCTA level.

Web Table 5: Adjusted Relative Risks of Firearm Self-Harm Among White Men Aged 50+

**Measure of Firearm Availability**	**Model by Exposure(s) Adjusted**[a]	**RR Overall (95% CI)**	**RR cPAR (95% CI)**
Firearm Dealers	Dealers & Alcohol	1.07 (1.03, 1.14)	1.03 (0.99, 1.07)
	Non-Pawn	1.05 (1.01, 1.10)	1.03 (1.00, 1.06)
	Pawn	1.01 (0.98, 1.04)	1.00 (0.97, 1.03)
	Off-Premise	1.00 (0.98, 1.01)	1.00 (0.99, 1.01)
	Bars/Pubs	1.01 (1.00, 1.02)	1.00 (0.99, 1.01)
Firearm Sales	Sales & Alcohol	1.17 (1.10, 1.26)	1.05 (1.03, 1.10)
	Sales	1.17 (1.09, 1.25)	1.05 (1.02, 1.09)
	Off-Premise	1.00 (0.99, 1.01)	1.00 (1.00, 1.00)
	Bars/Pubs	1.01 (1.00, 1.02)	1.00 (1.00, 1.01)

a. “Dealers” includes both non-pawn and pawn dealers. “Alcohol” includes both off-premise outlets and bars/pubs. Non-pawn dealers, pawn dealers, and firearm sales density were measured per 100,000 population at the county level. Off-premise outlets and bars/pubs were measured per 100,000 population at the ZCTA level.

Web Table 6: Sensitivity Analysis—Adjusted Relative Risks of Firearm Self-Harm, Excluding Long Guns

Measure of Firearm Availability	Model by Exposure(s) Adjusted[a]	RD Overall (95% CI)	RR Overall (95% CI)
Firearm Dealers	Dealers & Alcohol	0.01 (0.00, 0.03)	1.04 (0.99, 1.08)
	Non-Pawn	0.01 (-0.01, 0.02)	1.02 (0.98, 1.05)
	Pawn	0.00 (-0.01, 0.02)	1.01 (0.98, 1.04)
	Off-Premise	0.00 (0.00, 0.00)	1.00 (0.99, 1.00)
	Bars/Pubs	0.00 (0.00, 0.01)	1.01 (1.00, 1.02)
Firearm Sales	Sales & Alcohol	0.04 (0.02, 0.05)	1.09 (1.05, 1.14)
	Sales	0.03 (0.02, 0.05)	1.08 (1.04, 1.13)
	Off-Premise	0.00 (0.00, 0.00)	1.00 (1.00, 1.01)
	Bars/Pubs	0.00 (0.00, 0.01)	1.01 (1.00, 1.02)

a. "Dealers" includes both non-pawn and pawn dealers. "Alcohol" includes both off-premise outlets and bars/pubs. Non-pawn dealers, pawn dealers, and firearm sales density were measured per 100,000 population at the county level. Off-premise outlets and bars/pubs were measured per 100,000 population at the ZCTA level.
